# Unstable Distal Diametaphyseal Radius Fracture in Children a Retrospective Comparative Study of K-Wire Versus Plate Fixation

**DOI:** 10.3390/children13070922

**Published:** 2026-07-13

**Authors:** Chaojin Qin, Shi Gao, Xing Zhou, Haiqiong Chen, Xu Zhou, Guoqiang Zhao

**Affiliations:** Department of Orthopedic Trauma, Children’s Hospital, Zhejiang University School of Medicine, National Clinical Research Center for Children and Adolescents’ Health and Diseases, Hangzhou 310003, China; 6316087@zju.edu.cn (C.Q.); 6516021@zju.edu.cn (S.G.); 6515190@zju.edu.cn (X.Z.); chenhq10238@zju.edu.cn (H.C.); zhouxu9@zju.edu.cn (X.Z.)

**Keywords:** distal radius fracture, diametaphyseal, pediatric, plate fixation, K-Wire, forearm fracture index

## Abstract

**Highlights:**

**What are the main findings?**

- Plate fixation independently predicted superior alignment (adjusted OR = 14.48), whereas K-Wires’ faster early healing reflected lighter patient weight, not the implant.
- Bicortical K-Wires achieved optimal alignment among percutaneous options, whereas combined bicortical + intramedullary fixation yielded poor outcomes and should be avoided.

**What are the implications of the main findings?**

- In this cohort, older, heavier patients with higher FFI were more likely to undergo plate fixation. Both methods demonstrated low complication rates (3.2%).
- Due to selection bias and non-randomized allocation, these findings remain exploratory and require prospective validation.

**Abstract:**

**Background/Objectives**: Pediatric distal diametaphyseal radius fracture (DDRF) occurs in a challenging transitional zone between the metaphysis and diaphysis, presenting distinct biomechanical characteristics. This study compared outcomes of plate versus Kirschner wire (K-Wire) fixation—including different configurations—in a consecutive cohort of pediatric unstable DDRF. **Methods**: We conducted a single-center retrospective review of 63 patients (aged 6–15 years) treated between 2023 and 2025, divided into plate (*n* = 26) and K-Wire (*n* = 37) groups. The K-Wire cohort was subclassified as bicortical (*n* = 14), bicortical + intramedullary (*n* = 10), and intramedullary alone (*n* = 13). Outcomes included alignment quality, fracture healing by modified RUST (mRUST), and the Forearm Fracture Index (FFI). Multivariable regression was used to adjust for confounding. **Results**: Plate patients were older (11.92 ± 2.87 vs. 9.32 ± 2.96 years, *p* < 0.05) and heavier (43.23 ± 11.31 vs. 30.84 ± 11.19 kg, *p* < 0.001) and had higher FFI (median 1.13 vs. 1.09, *p* < 0.05). Multivariable regression showed plate fixation was independently associated with good alignment (adjusted OR = 14.48, *p* = 0.018). Bicortical K-Wires yielded the highest alignment rate among percutaneous techniques (85.7%), while bicortical + IM showed poor outcomes (0% good). The unadjusted 1-month healing advantage of K-Wires (mRUST 2.53 ± 0.60 vs. 2.19 ± 0.47, *p* = 0.022) was non-significant after adjusting for body weight (*p* = 0.248). The complication rate was low (3.2%, 2/63). At final follow-up (range 6–18 months), all patients demonstrated unrestricted wrist motion and forearm rotation, and functional outcomes were assessed clinically without validated patient-reported outcome measures. **Conclusions**: Plate fixation independently predicts superior alignment, whereas the K-Wire group’s higher 1-month mRUST scores were attributable to their younger, lighter profile rather than the implant itself. Among K-Wire options, bicortical configuration achieves reduction quality comparable to plates, while the bicortical + IM configuration should be avoided. In this cohort, plate fixation was more common among older, heavier patients with higher FFI, whereas younger, lighter patients more often received K-Wires; both methods demonstrated low complication rates.

## 1. Introduction

Distal forearm fractures represent the most common skeletal injuries in pediatric trauma, comprising approximately 20–25% of all childhood fractures and 74–85% of forearm fractures [1,2,3]. Among these, distal radius fractures located at the diametaphyseal junction—the transitional zone between the metaphysis and diaphysis—present distinct therapeutic challenges because of their unique biomechanical properties and healing patterns [4], accounting for approximately 16.1% of pediatric forearm fractures [5]. This region lacks the robust remodeling potential of the distal metaphysis and has insufficient diaphyseal stability for conventional intramedullary nailing techniques [6,7].

Closed reduction and casting remain the standard for stable distal radius fractures. However, unstable displaced diametaphyseal fractures show high redisplacement rates—up to 39% after conservative management. This often leads to unacceptable malunions and frequent re-operation [8,9,10]. The remodeling potential of pediatric bone, though substantial in younger children, diminishes significantly with age and is limited in the diametaphyseal region due to its distance from the physis [11,12]. Consequently, surgical stabilization is often required to prevent malunion, rotational deformity, and functional impairment [13].

Current surgical options for pediatric unstable distal diametaphyseal radius fractures include percutaneous Kirschner wire (K-Wire) fixation, elastic stable intramedullary nailing (ESIN), and open reduction with plate fixation [5,14]. K-Wire fixation is a minimally invasive option. It shortens operative time. When wires are left protruding, no second anesthesia is needed for removal [8,15]. However, stable fixation in the diametaphyseal region is technically challenging. The distal fragment is short, and the angle needed for bicortical purchase is steep. Risks include pin-site infection, wire migration, and secondary loss of reduction [16]. Recent modifications, including transepiphyseal intramedullary K-Wire techniques, have been proposed to address these limitations [15,17].

Conversely, plate fixation provides an alternative surgical option for pediatric unstable diametaphyseal radius fractures, particularly when accurate anatomical reduction is required [6,18,19]. This technique offers rigid internal fixation, allowing early mobilization and reliable restoration of radial alignment, which is especially valuable in older children with limited remodeling potential [11]. In pediatric patients, choosing the plating technique—specifically dorsal versus volar approaches—and implant positioning is critical. While volar plating is standard in adults [18], a dorsal approach is often favored for pediatric distal diametaphyseal radius fractures because it allows direct visualization of the dorsal cortex, facilitating accurate anatomical reduction in this transitional zone [6,19]. Regardless of the approach, meticulous positioning of the plate is paramount; screws must be placed strictly proximal to the active distal radial physis to prevent physeal violation and subsequent growth arrest [6,20]. This surgical positioning is highly dictated by the specific fracture location, as fractures closer to the physis demand greater technical precision to avoid physeal violation [6]. However, plate fixation involves greater surgical invasiveness compared to percutaneous techniques and typically requires a second procedure for implant removal in growing children [20,21].

ESIN represents another minimally invasive alternative for forearm fractures; nonetheless, technical challenges exist when applying this technique to the diametaphyseal region due to insufficient three-point fixation and risk of inadequate reduction [7,14,22,23]. Comparative evidence indicates that plate and K-Wire fixation may both outperform conventional ESIN in this region [6,19].

Despite the availability of these treatment modalities, controversy persists regarding the optimal fixation strategy for unstable distal diametaphyseal radius fractures, particularly concerning the relationship between fracture location, patient age, and optimal implant selection [24]. While several studies have examined metaphyseal or diaphyseal fractures separately, the diametaphyseal region represents a distinct entity with heterogeneous treatment responses [25]. Furthermore, the optimal K-Wire configuration (bicortical vs. IM alone vs. bicortical + IM) for this specific region remains undefined [5].

Therefore, the present retrospective study aims to compare the radiological outcomes (radiographic alignment and fracture healing assessed by modified RUST), complication rates, and perioperative parameters between plate fixation and various K-Wire fixation techniques in pediatric patients with unstable distal diametaphyseal radius fractures. We also sought to identify patient- and fracture-specific factors (including age, weight, and FFI) associated with treatment selection and outcomes. Given the observational design, we aimed to describe the outcomes associated with each fixation method without assuming causal superiority.

## 2. Materials and Methods

### 2.1. Study Design and Patient Population

**Figure 1:**
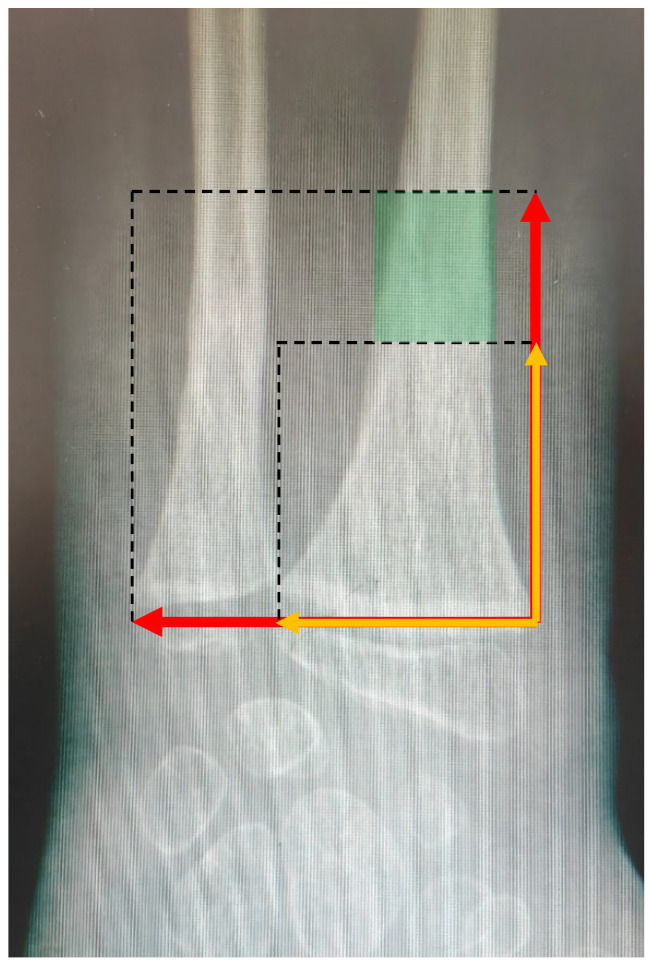
Definition of the distal diametaphyseal radius (green zone). Yellow arrow: radial physeal width; Red arrow: combined radial and ulnar physeal widths.

We performed a retrospective comparative study of pediatric patients treated for unstable distal diametaphyseal radius fractures at the Children’s Hospital, Zhejiang University, School of Medicine, between 2023 and 2025. Institutional review board approval was obtained, and informed consent was waived due to the retrospective nature of the study. The forearm fracture index (FFI) was calculated on radiographs as follows: (distance from fracture to distal radial physis)/(width of radial physis). This method has been described by Schrottenberg et al. [26]. The distal diametaphyseal radius was defined as the transitional zone proximal to the metaphysis, as shown in Figure 1. Patients were included if they met all of the following criteria: (1) age < 16 years, (2) distal radius fracture located within the distal diametaphyseal radius zone, (3) unstable fracture pattern requiring surgical stabilization (complete displacement, >15° angulation, or failure of closed reduction), and (4) minimum 6-month clinical follow-up. Exclusion criteria included open fractures, pathological fractures, ipsilateral upper extremity concomitant injuries, previous forearm fractures, closed distal radial physes, and loss to follow-up.

Because treatment was determined by surgeon judgment based on individual patient and fracture characteristics rather than randomization, the two groups are not directly comparable for causal inference. Accordingly, this study is descriptive in nature, focusing on observed associations between fixation method and clinical outcomes, with the aim of informing future prospective studies.

### 2.2. Surgical Techniques

Patients underwent either dorsal plate fixation or K-Wire fixation based on surgeon preference and patient characteristics.

**Figure 2:**
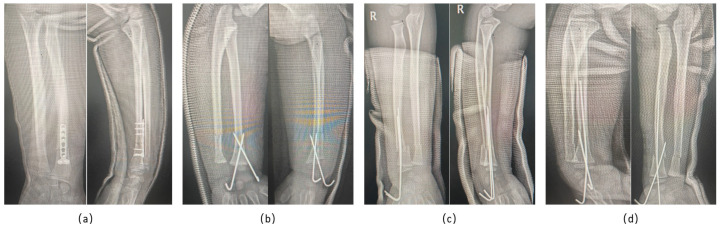
Representative postoperative radiographs illustrating the four surgical techniques. (**a**) Plate fixation. (**b**) Bicortical fixation. (**c**) Intramedullary (IM) alone fixation. (**d**) Combined bicortical-plus-intramedullary (bicortical + IM) fixation.

For plate fixation, a dorsal approach was utilized for exposure of the distal radius fracture. After open anatomic reduction under direct visualization, a low-profile dorsal locking plate (1.5 mm thickness, Double Medical Technology Inc., Xiamen, China) with 2.7 mm locking screws was applied. Locking screws were placed strictly proximal to the physis to avoid physeal injury. The wound was closed in layers after hemostasis. For K-Wire fixation, three K-Wire fixation techniques were employed: (1) bicortical fixation: two K-Wires inserted through the radial styloid process or epiphysis, anchored in the contralateral cortex; (2) intramedullary (IM) alone fixation: a K-Wire advanced through the medullary canal after reduction; (3) combined bicortical-plus-intramedullary (bicortical + IM) fixation: both bicortical and intramedullary wires were used for augmented stability. All wires were left percutaneously protruding for subsequent outpatient removal. Representative postoperative radiographs of each technique are shown in Figure 2.

### 2.3. Radiographic Assessment and Outcome Measures

Standardized anteroposterior and lateral radiographs were obtained preoperatively, immediately postoperatively, and at 1- and 2-month follow-ups for radiographic union assessment. Postoperative reduction alignment was evaluated by measuring angular deformity in the coronal and sagittal planes, which was graded as follows: Good (<5°), Moderate (5–10°), and Poor (>10°) [5]. Fracture healing was assessed using the modified Radiographic Union Score for Tibial fractures (mRUST) [27]. Although originally developed for tibial fractures, the mRUST system evaluates fundamental biological hallmarks of healing (callus formation and bicortical bridging) that are common to all long bones. The clinical utility and reliability of RUST-based scoring have been previously validated for forearm fractures by Kizkapan et al. [28]. Because the dorsal plate obscured the dorsal cortex, plate fixation allowed visualization of only three of the four standard cortices, whereas K-Wire fixation permitted assessment of all four. To ensure inter-group comparability, mRUST scores were normalized by averaging across assessable cortices for both groups (range 1.0–3.0). For intra-group comparison among K-Wire subgroups, the standard four-cortex total score was utilized. To minimize interobserver variability, two independent orthopedic surgeons performed all assessments, blinded to treatment allocation and to each other’s scores; discrepancies were resolved by consensus. Formal interobserver reliability (ICC or kappa) was not calculated, which is a limitation of this study.

Primary outcomes included radiographic reduction quality and fracture union time. Secondary outcomes included operation time, length of hospital stay, open reduction rate, and postoperative complications, which were graded using the modified Clavien–Dindo classification [29,30].

### 2.4. Postoperative Management

Postoperatively, patients in the K-Wire group were immobilized in an above-elbow plaster cast for 6 to 8 weeks, whereas patients in the plate group were immobilized in a plaster splint or below-elbow cast for 4 to 6 weeks. K-Wires were removed in the outpatient clinic at 6–8 weeks when radiographic callus formation was observed. For patients treated with plate fixation, implant removal was performed electively at 6 months postoperatively to minimize refracture risk. Functional rehabilitation including wrist and forearm range-of-motion exercises was initiated after the end of the immobilization.

### 2.5. Statistical Analysis

Normality was assessed using the Shapiro–Wilk test. Normally distributed continuous variables are reported as mean ± SD and compared by Student’s *t*-test; non-normally distributed variables as median (IQR) and compared by the Mann–Whitney U test; categorical variables as frequencies (%) and compared by Fisher’s exact test. For comparisons among K-Wire subgroups, continuous variables were compared by the Kruskal–Wallis test and categorical variables by Fisher’s exact test. Statistical significance was set at *p* < 0.05.

To adjust for baseline imbalances, multivariable logistic regression was performed to evaluate the independent association between fixation method and radiographic alignment (good vs. moderate/poor reduction), adjusting for age, weight, FFI, and sex. Firth’s bias-reduced regression addressed quasi-complete separation, and 1:1 nearest-neighbor propensity score matching (PSM) was conducted as a sensitivity analysis. For longitudinal mRUST scores, multivariable linear regression was performed at each time point, and a linear mixed-effects model (LMM) with a random intercept analyzed healing trajectories. All analyses were performed using SPSS version 26.0 (IBM Corp., Armonk, NY, USA) and the R software environment version 4.3.1 (using ‘logistf’ and ‘forestplot’ packages).

## 3. Results

### 3.1. Demographic and Clinical Characteristics

A total of 63 pediatric patients with unstable distal diametaphyseal radius fractures were included in this retrospective study. The patients were divided into two main groups: the K-Wire fixation group (*n* = 37) and the plate fixation group (*n* = 26). The demographic and clinical characteristics of both groups are summarized in Table 1.

There was a significant difference in age between the two groups, with patients in the plate group being older (11.92 ± 2.87 years) compared to the K-Wire group (9.32 ± 2.96 years) (*p* < 0.05). Similarly, body weight was significantly higher in the plate group (43.23 ± 11.31 kg) than in the K-Wire group (30.84 ± 11.19 kg) (*p* < 0.001). However, no significant difference was observed in sex distribution between the two groups (*p* = 0.73).

Regarding surgical parameters, the operation time was significantly shorter in the K-Wire group (51.89 ± 18.24 min) compared to the plate group (65.92 ± 18.4 min) (*p* < 0.05). The FFI was significantly higher in the plate group (1.13 [1.07–1.25]) than in the K-Wire group (1.09 [1.05–1.13], *p* < 0.05). Hospital stay was significantly longer for patients in the plate group (7.65 ± 1.94 days) compared to the K-Wire group (5.05 ± 1.67 days) (*p* < 0.001). Regarding the surgical approach, all patients (100%) in the plate group underwent open reduction, while the open reduction rate was 56.76% (21/37) in the K-Wire group.

### 3.2. Demographic Characteristics of K-Wire Subgroups

The K-Wire fixation group was further subdivided into three categories based on the fixation technique: bicortical fixation (*n* = 14), bicortical + IM fixation (*n* = 10), and IM alone fixation (*n* = 13). The demographic characteristics of these subgroups are presented in Table 1.

No significant differences were observed among the three subgroups in terms of age (*p* = 0.136), sex distribution (*p* = 0.344), operation time (*p* = 0.864), fracture index (*p* = 0.090), hospital stay (*p* = 0.577) or open reduction rate (*p* = 0.22). However, body weight showed significant differences among the subgroups (*p* = 0.031), with the bicortical group having the highest mean weight (36.6 ± 15.1 kg) compared to the bicortical + IM group (25.2 ± 7.2 kg) and the IM alone group (28.6 ± 5.2 kg).

### 3.3. Postoperative Reduction Quality and Radiological Assessment

Postoperative radiological outcomes, including angular deformity and fracture healing (assessed with mRUST), are summarized in Table 2.

A higher proportion of patients in the plate group achieved good reduction compared to the K-Wire group (96.2% vs. 59.5% good reduction; *p* < 0.001). At the 1-month postoperative follow-up, the K-Wire group showed significantly higher mRUST scores compared to the plate group (2.53 ± 0.60 vs. 2.19 ± 0.47, *p* = 0.022). However, at the 2-month follow-up, the difference in mRUST scores between the two groups was no longer statistically significant (3.48 ± 0.51 vs. 3.31 ± 0.65, *p* = 0.161).

Among K-Wire subgroups, alignment outcomes varied considerably. Bicortical fixation achieved good reduction in 85.7% of cases, statistically comparable to plate fixation (85.7% vs. 96.2%; *p* = 0.25). IM alone fixation demonstrated good alignment in 76.9% of cases. In contrast, the bicortical + IM subgroup exhibited poor alignment outcomes with no cases achieving good reduction (0% good, 80% moderate, 20% poor). Bicortical + IM exhibited significantly worse alignment compared to both Bicortical (0% vs. 85.7%) and IM alone (0% vs. 76.9%; both *p* < 0.01). No significant differences were observed among the K-Wire subgroups at either 1 month or 2 months (*p* > 0.05).

### 3.4. Complications and Functional Outcomes

At final follow-up (ranging from 6 to 18 months postoperatively), all patients demonstrated unrestricted wrist motion and forearm rotation without functional limitations. Clinical evaluation revealed full recovery of pronation, supination, wrist flexion, and extension in all cases. No patient or parent reported residual difficulties with activities of daily living, including writing, grasping objects, or participating in sports.

The overall complication rate was low at 3.2% (2/63), with both cases classified as mild complications (Clavien-Dindo Grade I) [29,30]. In the K-Wire group, one patient (2.7%) experienced a refracture of the distal radius 6 months after premature implant removal (performed at 8 weeks postoperatively). This 11-year-old male sustained a low-energy fall resulting in a refracture with acceptable alignment at the previous fracture site. The refracture was successfully managed with closed reduction and above-elbow cast immobilization for 6 weeks, followed by functional bracing. At final follow-up, the patient demonstrated full union with excellent clinical function and no residual deformity.

In the plate group, one patient (3.8%) exhibited delayed union requiring prolonged cast immobilization for 3 months (rather than the conventional 4–6 weeks). The fracture eventually achieved clinical and radiographic union without additional intervention. No cases of superficial or deep infection, pin-site infection, tendon rupture, neurovascular injury, or physeal growth disturbance were observed in either group.

### 3.5. Multivariable Analysis

#### 3.5.1. Logistic Regression for Radiographic Alignment

**Figure 3:**
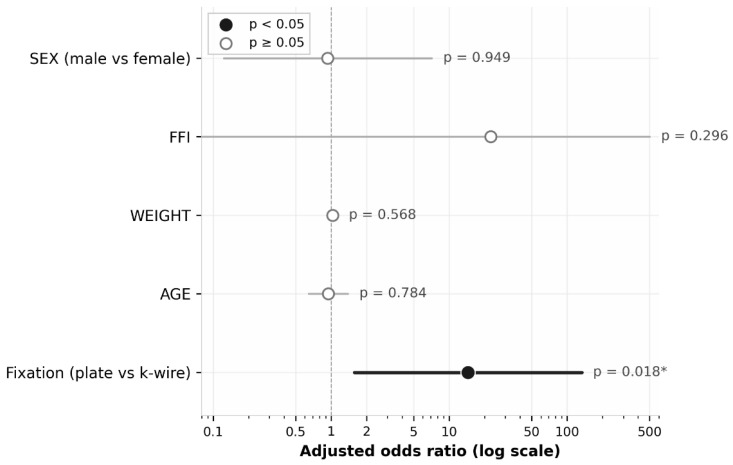
Forest plot of multivariable logistic regression for radiographic alignment (*n* = 63). Black-filled circles indicate *p* < 0.05; open circles indicate *p* ≥ 0.05; The asterisk (*) denotes statistical significance. The dashed vertical line represents the null association (OR = 1).

Multivariable logistic regression adjusting for age, weight, FFI, and sex was performed (Table 3). Plate fixation remained independently associated with good alignment (Figure 3; aOR = 14.48, 95% CI: 1.57–133.32, *p* = 0.018). No other covariates reached statistical significance. Sensitivity analyses using Firth’s bias-reduced logistic regression and 1:1 propensity score matching (PSM, *n* = 52) yielded consistent results (OR = 14.47 and 21.43, respectively; both *p* < 0.05). Tests for effect modification by age, weight, and FFI were all non-significant (all *p* > 0.05).

#### 3.5.2. Linear Regression for mRUST

At 1 month, multivariable linear regression showed no significant independent association between fixation method and mRUST scores (β = −0.189, 95% CI: −0.512 to 0.135, *p* = 0.248). Body weight demonstrated the largest effect size among covariates but remained non-significant (β = −0.018 per kg, 95% CI: −0.037 to 0.002, *p* = 0.070). At 2 months, neither fixation method nor any covariate was significantly associated with healing scores (fixation: β = −0.117, *p* = 0.499). A linear mixed-effects model confirmed significant healing progression over time (β = 0.946, *p* < 0.001) but no significant Time × Fixation interaction (β = 0.169, *p* = 0.273), indicating comparable healing trajectories between groups after adjustment.

## 4. Discussion

The treatment of unstable distal diametaphyseal radius fracture (DDRF) in children remains a subject of ongoing debate in pediatric orthopedics. This retrospective study compared two fixation methods—plate fixation and K-Wire—in a cohort of children with DDRF. Given the observational design, our findings provide descriptive insights into the observed associations between treatment strategies, patient characteristics, and radiological outcomes.

### 4.1. Patient Demographics and Treatment Selection

Our results revealed significant differences in age and body weight between the two groups, with patients in the plate fixation group being older and heavier. Greig et al. [11]. similarly found that adolescents near skeletal maturity (typically >12 years) more often undergo plate fixation. In the study by van Egmond et al. [18], the median age of children treated with volar plate fixation was 12.5 years, which closely matches the mean age of our plate group (11.92 years). These findings indicate that surgeons tend to select plate fixation for older and heavier children to achieve both anatomic precision and enhanced mechanical stability [12].

Beyond patient age and body weight, the anatomical location of the fracture within the diametaphyseal zone represents a fundamental determinant of treatment selection. The FFI revealed important insights regarding fracture location and treatment selection. In our cohort, the plate group exhibited a significantly higher FFI, indicating the fracture line is positioned further from the distal radial physis, compared to the K-Wire group. Although FFI was not an independent predictor of alignment, its baseline difference suggests surgeons prefer plate fixation for proximal fractures (higher FFI) and percutaneous techniques for physeal-near fractures (lower FFI). Therefore, FFI serves as a valuable clinical decision-making parameter reflecting real-world treatment selection [26].

These selection criteria manifest in distinct surgical approaches and perioperative parameters. The mean operative time in the K-Wire group was significantly shorter than that in the plate group. This finding is consistent with previous reports on pediatric diametaphyseal radius fractures [6]. Similarly, Zheng et al. [19] found that K-Wire fixation required significantly less operative time than plate fixation in older children. Prolonged operative time increases complication risk in pediatric fractures [13]. This supports the clinical efficiency of K-Wire fixation.

These intraoperative differences translated into longer hospital stays for the plate group (7.65 vs. 5.05 days). This difference likely relates to the extent of surgical trauma and postoperative management protocols. The prolonged hospitalization in both groups reflects our institutional protocol, where preoperative evaluation, postoperative observation, and discharge readiness assessment are routinely incorporated. This prolonged stay represents an institutional-level factor rather than a surgical necessity and does not affect the primary outcomes under comparison. Wang et al. [17] reported that open reduction and internal fixation involved longer operative times and larger incisions, resulting in greater surgical trauma and typically requiring longer postoperative recovery and hospitalization compared to K-Wire fixation. Additionally, plate removal requires a second admission and anesthesia, increasing the hospitalization burden.

### 4.2. Reduction Quality and Radiological Outcomes

Different fixation strategies yielded distinct reduction outcomes. Multivariable analysis confirmed that plate fixation was independently associated with superior alignment (aOR = 14.48, *p* = 0.018), underscoring the mechanical advantage of rigid internal fixation at the diametaphyseal junction. However, the wide 95% confidence interval (1.57–133.32) reflects a limited sample size (*n* = 63) and cautions against overinterpretation; this finding requires validation in larger cohorts. The 56.8% open reduction rate reflects the technical difficulty of closed reduction in the short distal fragment; in selected cases, limited open exposure was employed to avoid repeated percutaneous attempts and minimize physeal injury, while still preserving substantially less surgical trauma than formal plate fixation. Among K-Wire subgroups, the bicortical + IM configuration yielded 0% good alignment—significantly worse than both bicortical (85.7%) and IM alone (76.9%) (both *p* < 0.01). This uniform failure raises concern about biomechanical incompatibility when combining divergent fixation principles in the diametaphyseal zone. However, given the small subgroup size (*n* = 10), this finding should be considered exploratory and interpreted with caution. Larger studies are needed before definitive clinical recommendations can be made. In contrast, bicortical K-Wire fixation achieved alignment comparable to plate fixation (85.7% vs. 96.2%, *p* = 0.25) and represents a viable percutaneous alternative.

Alternatively, IM alone fixation represents a pragmatic trade-off: its anatomical accuracy is modestly inferior to bicortical fixation, but the lower open reduction rate (38.5% vs. 71.4%) preserves fracture site biology. Given equivalent healing kinetics among K-Wire subgroups, this compromise may be acceptable in skeletally immature patients. Nevertheless, IM alone fixation requires transepiphyseal pin placement; surgeons must weigh this benefit against the risk of iatrogenic physeal injury.

K-Wire fixation showed higher 1-month mRUST scores (2.53 vs. 2.19, *p* = 0.022) in an unadjusted comparison. This advantage disappeared after multivariable adjustment (β = −0.189, *p* = 0.248). Body weight showed a borderline association (*p* = 0.070) (*p* = 0.07). This reversal illustrates confounding by indication: the K-Wire group comprised younger, lighter patients whose intrinsic healing capacity—not the implant itself—drove the apparent early advantage. Clinically, perceived differences in healing speed should not influence implant selection; patient physiology (particularly weight) is the dominant driver of early radiographic union. By two months, no significant differences in mRUST scores were observed between fixation groups, indicating that the initial unadjusted disparity attributable to baseline patient characteristics (particularly lower body weight in the K-Wire cohort) had resolved over time. Among K-Wire subgroups, equivalent mRUST scores at both timepoints further support that percutaneous configurations yield comparable healing trajectories once initial stability is achieved [5]. Consequently, these findings support a practical decision framework: dorsal plating is suitable for older, heavier patients with proximal fractures requiring rigid stabilization (high FFI), whereas younger, lighter patients may be treated with percutaneous pinning (bicortical for stability, intramedullary to minimize trauma), avoiding the hybrid construct. All K-Wire techniques here inevitably cross the physis. Physeal-sparing alternatives exist but offer limited purchase in the short diametaphyseal segment. Available evidence indicates smooth transphyseal K-Wires carry minimal growth risk [5].

### 4.3. Complications and Safety Considerations

In our cohort, the overall complication rate was low at 3.2% (2/63) during follow-up (range 6–18 months). One patient had a refracture (K-Wire group). One had a delayed union (plate group). Both were Clavien-Dindo grade I. No infections, neurovascular injuries, or physeal disturbances were observed in either group, attesting to the safety of both approaches in experienced hands.

Although volar locking plates represent the mainstream approach for distal radius fractures in adults [18], we selected a dorsal approach with 1.5 mm low-profile locking plates to facilitate direct visualization of the dorsal cortex and precise reduction in this transitional zone [31]. Modern low-profile designs (1.2–1.6 mm) with rounded edges and flush screw heads significantly reduce the soft-tissue complications historically associated with thicker dorsal implants (20–30% extensor tendon irritation) [32,33]. At the same time, they maintain adequate mechanical stability. All dorsal plates were routinely removed at 6 months postoperatively, a threshold supported by Weil et al. [6], to minimize refracture risk in this anatomical region. Proactive removal was elected to prevent potential growth-related complications in this pediatric cohort [20,21]. Notably, even volar locking plates have demonstrated a 58% removal rate in pediatric series [18]. One patient (3.8%) in the plate group exhibited delayed union requiring prolonged immobilization (three months), eventually achieving union without additional intervention. While our statistical analysis shows equivalent overall healing rates between the two cohorts, this individual case highlights the potential biological cost of open reduction. Disruption of the periosteal blood supply and local surgical trauma can occasionally delay healing in specific patient subgroups. In this regard, percutaneous K-Wire fixation aligns with the principle of tissue preservation, as emphasized by Fulchignoni et al. [34]. Furthermore, preserving local soft tissues and the fracture hematoma maintains a favorable microenvironment rich in osteogenic factors, a concept closely related to the regenerative potential and tissue preservation strategies discussed by Bonetti et al. [35]. Ultimately, the robust osteogenic potential of the pediatric skeleton prevents these localized biological costs from resulting in a systemic difference in final union times. In terms of functional recovery, all children in our cohort eventually regained a full range of motion of the wrist and forearm, returning to their pre-injury activity levels. However, we acknowledge that the absence of standardized PROMs limits our ability to detect subtle patient-perceived deficits, despite the generally excellent functional recovery typical of pediatric distal radius fractures. This common limitation in retrospective series highlights the need for future prospective studies incorporating PROMs to validate these findings. The absence of infections or neurovascular complications in our series supports the safety of both approaches, but underscores the necessity of meticulous surgical technique. While the 6–18-month follow-up captured fracture union and early complications, it may be insufficient to exclude late-onset physeal disturbances following transepiphyseal pinning, as highlighted by Mancini et al. [36].

### 4.4. Limitations

This study has several limitations. First, the retrospective, non-randomized design introduces selection bias. Second, the relatively small sample size (*n* = 63) from a single center limits the statistical power for K-Wire subgroup comparisons. Third, interobserver reliability for the adapted mRUST scoring was not formally assessed. Fourth, the 6–18-month follow-up may be insufficient to detect late-onset physeal disturbances after transepiphyseal pinning. Therefore, no causal conclusions regarding implant superiority can be drawn, and these findings remain exploratory and hypothesis-generating.

## 5. Conclusions

In conclusion, this study demonstrates that plate fixation independently predicts superior alignment (aOR = 14.48) in pediatric unstable DDRF, whereas the apparent early healing advantage of K-Wire fixation is likely confounded by baseline patient characteristics (particularly body weight) rather than the implant itself. Among percutaneous options, the bicortical K-Wire configuration achieves reduction quality comparable to plates, whereas the hybrid bicortical + IM configuration yields poor outcomes and should be avoided in this cohort. Furthermore, patient-specific factors (age, weight, FFI) are strongly associated with treatment selection, supporting personalized surgical planning. Both methods demonstrate a favorable safety profile with low complication rates.

## Figures and Tables

**Table 1:** Demographic and clinical characteristics.

Parameter	Plate (*n* = 26)	K-Wire (*n* = 37)				*p* Value *
		Total (*n* = 37)	Bicortical (*n* = 14)	Bicortical + IM (*n* = 10)	IM Alone (*n* = 13)	
Age (yrs)	11.92 ± 2.87	9.32 ± 2.96	10.4 ± 3.5	8.0 ± 2.5	9.2 ± 2.4	<0.05
Sex (Male/Female), *n*	23/3	31/6	12/2	7/3	12/1	0.73
Weight (kg) †	43.23 ± 11.31	30.84 ± 11.19	36.6 ± 15.1	25.2 ± 7.2	28.6 ± 5.2	<0.001
Operation time (min)	65.92 ± 18.4	51.89 ± 18.24	54.1 ± 18.6	49.8 ± 18.8	51.2 ± 18.7	<0.05
Forearm fracture index (FFI)	1.13 (1.07–1.25)	1.09 (1.05–1.13)	1.06 (1.04–1.12)	1.09 (1.05–1.11)	1.12 (1.09–1.25)	<0.05
Hospital stay (days)	7.65 ± 1.94	5.05 ± 1.67	5.0 (4.0–7.0)	5.5 (4.0–6.0)	5.0 (4.0–6.0)	<0.001
Open reduction rate, *n* (%)	26 (100)	21 (56.76)	10 (71.43)	6 (60)	5(38.46)	— §

* K-Wire groups (total) combined vs. Plate group; † Weight differed significantly among K-Wire subgroups (*p* = 0.031); All patients in the plate group underwent open reduction (100%); § Formal statistical comparison not performed for open reduction rate: plate fixation inherently requires open reduction (100% by definition); other parameters showed no significant differences among subgroups (all *p* > 0.05); IM, Intramedullary.

**Table 2:** Postoperative radiological assessment.

Parameter		Plate (*n* = 26)	K-Wire (*n* = 37)				*p* Value ‡
			Total (*n* = 37)	Bicortical (*n* = 14)	Bicortical + IM (*n* = 10)	IM Alone (*n* = 13)	
Alignment (*n*%)	Good	25 (96.2)	22 (59.5)	12 (85.7)	0 (0)	10 (76.9)	<0.001
	Moderate	1 (3.8)	11 (29.7)	2 (14.3)	8 (80)	1 (7.7)	
	Poor	0 (0)	4 (10.8)	0 (0)	2 (20)	2 (15.4)	
mRUST score at 1 month, mean ± SD		2.19 ± 0.47	2.53 ± 0.60	9.8 ± 2.0	10.6 ± 3.1	10.2 ± 2.4	0.022
mRUST score at 2 months, mean ± SD		3.31 ± 0.65	3.48 ± 0.51	13.6 ± 2.0	13.9 ± 2.6	14.3 ± 1.8	0.161

‡ Plate vs. K-Wire (total). mRUST: mean cortex scores for inter-group comparison; total scores (4 cortices) for K-Wire subgroups. Inter-subgroup comparisons are described in the text.

**Table 3:** Predictors of radiographic alignment (good vs. moderate or poor reduction): univariate and multivariable logistic regression analysis.

Parameter	Univariate		Multivariable	
	OR (95% CI)	*p* Value	aOR (95% CI)	*p* Value
Fixation (plate vs. K-Wire)	19.0 (3.0–121.6)	<0.001	14.48 (1.57–133.32)	0.018
Age (per year)	1.18 (0.97–1.43)	0.091	0.95 (0.65–1.39)	0.784
Weight (per kg)	1.06 (1.00–1.12)	0.039	1.03 (0.93–1.13)	0.568
Forearm Fracture Index (FFI)	76.9 (0.32–∞) †	0.121	22.47 (0.07–7747.81)	0.296
SEX (male vs. female)	1.43 (0.41–5.03)	0.643	0.94 (0.12–7.14)	0.949

Univariate estimates are provided for descriptive reference only. † Upper confidence limit for FFI exceeded computational bounds due to quasi-complete separation. Multivariable model fit: Likelihood ratio χ^2^ = 15.96, *p* = 0.007; Nagelkerke R^2^ = 0.325; Hosmer–Lemeshow test *p* = 0.789.

## Data Availability

The datasets used and/or analyzed during the current study are not publicly available due to patient privacy and confidentiality regulations, but are available from the corresponding author on reasonable request.

## Abbreviations

The following abbreviations are used in this manuscript:

DDRF	Distal Diametaphyseal Radius Fractures
FFI	Forearm Fracture Index
mRUST	modified Radiographic Union Score for Tibial Fractures
IM	Intramedullary
